# Pathophysiology and clinical implications of dengue-associated neurological disorders

**DOI:** 10.3389/fmicb.2025.1536955

**Published:** 2025-09-12

**Authors:** Ramtin Naderian, Elham Paraandavaji, Amir Hossein Maddah, Saeedeh Keshavarzi, Anoosha Habibian, Rayan Naderian, Seyed Mohammad Hosseini, Valentyn Oksenych, Majid Eslami

**Affiliations:** ^1^Universal Scientific Education and Research Network (USERN), Semnan, Iran; ^2^Nervous System Stem Cells Research Center, Semnan University of Medical Sciences, Semnan, Iran; ^3^Skull Base Research Center, Loghman Hakim Hospital, Shahid Beheshti University of Medical Sciences, Tehran, Iran; ^4^Student Research Committee, School of Medicine, Shahroud University of Medical Sciences, Shahroud, Iran; ^5^Department of Pediatrics, School of Medicine, Semnan University of Medical Sciences, Semnan, Iran; ^6^University of Bergen, Bergen, Norway; ^7^Department of Clinical and Molecular Medicine, Norwegian University of Science and Technology (NTNU), Trondheim, Norway; ^8^Department of Biosciences and Nutrition, Karolinska Institutet, Huddinge, Sweden; ^9^Department of Bacteriology and Virology, Faculty of Medicine, Semnan University of Medical Sciences, Semnan, Iran

**Keywords:** dengue, DENV, encephalitis, neuroinvasion, neurological disorder

## Abstract

Dengue virus (DENV), a mosquito-borne *Flavivirus*, represents a growing global health challenge, particularly in tropical and subtropical regions. The 2009 WHO classification system for dengue categorizes infections into dengue without warning signs, dengue with warning signs, and severe dengue. This framework highlights the diverse clinical presentations and supports more efficient triage and management of cases. The neurological effects of DENV infection, which include direct neuroinvasion, systemic problems, and immune-mediated sequelae, are a less well-studied but nevertheless important consequence. Guillain-Barré syndrome, acute disseminated encephalomyelitis, myelitis, meningitis, and encephalitis are important neurological symptoms. The dengue classification system improves clinical management by dividing cases into dengue without warning signs, dengue with warning signs, and severe dengue. Mild cases show fever with symptoms like headache and rash, while warning signs include abdominal pain, persistent vomiting, bleeding, and lab changes indicating higher risk. Severe dengue is characterized by critical complications such as shock, severe bleeding, or organ failure. Improved diagnostics aid early detection, and new treatments targeting viral replication and inflammation are being explored alongside supportive care. Although there are still challenges in reaching the ideal vaccination coverage, the introduction of potent vaccines like Dengvaxia and Qdenga provide an achievable option for prevention. Thorough study into DENV’s neurological effects and treatment options is essential as the virus’s geographic range is increased by climate change and international travel. Reducing the worldwide burden of dengue-related neurological complications requires addressing the intricate interactions between virological, immunological, and environmental variables.

## 1 Introduction

Dengue virus (DENV) is a positive-sense RNA virus with a genome size of approximately 10.7 Kb, belonging to the *Flaviviridae* family (Chambers et al., 1990; Westaway et al., 1985). DENV is primarily transmitted by *Aedes aegypti* and *Aedes albopictus* mosquitoes, although it can also be transmitted by *Aedes vitattus.* The global endemicity of DENV is a result of this widespread vector range, particularly in tropical and subtropical regions (Lee et al., 2018). In 2009, the World Health Organization (WHO) revised its dengue classification system to enhance clinical management and improve case identification, replacing the older 1997 model. The previous approach classified cases into dengue fever, dengue hemorrhagic fever, and dengue shock syndrome. The updated system focuses on identifying warning signs and categorizes dengue into three main groups: dengue without warning signs, dengue with warning signs, and severe dengue. Dengue without warning signs generally refers to febrile illness accompanied by symptoms such as headache, rash, muscle and joint pain, nausea, and mild bleeding. Dengue with warning signs includes additional clinical indicators signaling a greater risk of progression to severe disease. These warning signs may include persistent abdominal pain, continuous vomiting, fluid buildup, mucosal bleeding, lethargy, liver enlargement, and laboratory findings such as a rising hematocrit level coupled with a rapid drop in platelet count. Severe dengue is marked by significant plasma leakage leading to shock or respiratory distress due to fluid accumulation, severe bleeding, or critical organ impairments affecting the liver, central nervous system, or heart. This updated classification aims to facilitate early detection of cases with severe potential, ensuring timely interventions and reducing mortality rates (WHO Guidelines Approved by the Guidelines Review Committee, 2009). Notably, because DENV may enter the central nervous system (CNS) and cause encephalitis, meningitis, and myelitis, neurological involvement has attracted more attention. Neurological symptoms such as altered consciousness, neck rigidity, focal neurological signs, and convulsions are observed in approximately 5% of cases (Verma et al., 2013; Murthy, 2010).

Dengue virus infection progresses through three distinct phases: the febrile phase, the critical phase, and the recovery phase, each characterized by specific virological and immunological dynamics (Martina et al., 2009). The febrile phase typically starts 4–7 days after a mosquito bite and is marked by high fever, headache, muscle pain, and laboratory findings of viremia. Viremia tends to peak early, usually declining by days 4 or 5 of illness as the host’s adaptive immune system activates and seroconversion occurs. Viral RNA is generally cleared from the bloodstream between days 5 and 7 but may persist longer in cerebrospinal fluid or other immune-privileged sites (Soares et al., 2006). The critical phase, overlapping with defervescence (usually around days 4–6), presents the greatest clinical risks. During this period, patients may experience plasma leakage, thrombocytopenia, and hemorrhagic complications. Neurological symptoms can also develop during or shortly after this phase, depending on the underlying mechanisms. Complications such as encephalopathy and encephalitis may arise early in severe cases, likely resulting from direct viral invasion or cytokine-driven neuroinflammation (Solomon et al., 2000). In contrast, delayed immune-mediated conditions like Guillain-Barré syndrome, acute disseminated encephalomyelitis (ADEM), or transverse myelitis generally appear 1–3 weeks following the acute febrile stage, indicative of post-infectious autoimmune responses (Murthy, 2010; Carod-Artal et al., 2013). Additionally, some patients may develop post-acute complications, including prolonged fatigue, cognitive difficulties, or mood disturbances collectively referred to as post-dengue syndrome which can persist for weeks or even months. This underscores the importance of not only acute-phase care but also long-term monitoring for neurological and neuropsychiatric sequelae in those affected (Sigera et al., 2021).

Accurate and timely diagnosis of dengue virus infection plays a critical role in preventing disease progression and associated complications. Current diagnostic strategies include serological tests such as NS1 antigen detection and IgM/IgG ELISA, molecular techniques like RT-PCR, as well as neuroimaging methods like MRI for patients exhibiting neurological symptoms. These tools are particularly vital in atypical presentations, where dengue manifests with conditions such as encephalopathy, stroke, or peripheral neuropathies instead of the typical febrile illness (Hunsperger et al., 2014; Lnu et al., 2022). The global impact of dengue has risen sharply in recent decades due to factors like climate change, urbanization, population expansion, and the broader distribution of Aedes mosquito habitats (Messina et al., 2014). Furthermore, international travel and inadequate vector control measures in endemic areas have contributed to the resurgence and simultaneous circulation of multiple serotypes, often leading to hyperendemicity and heightened risk of severe disease outcomes. Within the diverse clinical spectrum of dengue, neurological complications are increasingly recognized but remain frequently underdiagnosed. Research indicates that up to 21% of severe dengue cases may involve neurological symptoms, ranging from mild issues like headache and dizziness to more serious conditions such as encephalitis, Guillain-Barré syndrome, acute transverse myelitis, and ADEM. This underscores the necessity for integrated diagnostic approaches and surveillance systems that acknowledge the virus’s ability to impact the nervous system (Murthy, 2010).

Despite significant research efforts, no specific antiviral treatment has yet been developed. As a result, managing the disease remains highly dependent on supportive care and preventive measures, with a strong emphasis on vector control and public health interventions (Guzman et al., 2016). In the fight against dengue fever (DF), recent developments in vaccine development have produced two licensed vaccines, Dengvaxia and Qdenga. While Qdenga, a more recent addition, may help prevent diseases across a wider age range and serostatus, Dengvaxia, a live-attenuated tetravalent vaccination, has demonstrated effectiveness in seropositive persons (WHO Guidelines Approved by the Guidelines Review Committee, 2009).

Despite extensive global efforts, no specific antiviral therapy has been approved to treat dengue virus infections to date. As a result, management remains largely supportive. In response, vaccination has taken center stage in dengue prevention strategies, especially in regions where the disease is most prevalent. Two vaccines, Dengvaxia (CYD-TDV) and Qdenga (TAK-003) have emerged as significant breakthroughs in this field, each characterized by unique immunogenicity profiles, efficacy outcomes, and practical limitations. Dengvaxia (CYD-TDV), developed by Sanofi Pasteur, is a live attenuated, recombinant tetravalent vaccine. It is based on a yellow fever virus (YF17D) backbone, incorporating the premembrane (prM) and envelope (E) genes of the four dengue virus serotypes (DENV-1 to DENV-4). Clinical trials have shown that it provides moderate to high efficacy among individuals with prior dengue exposure, particularly against serotypes DENV-3 and DENV-4, though its effectiveness is lower against DENV-1 and DENV-2 (Sridhar et al., 2018). However, post-licensure studies revealed that Dengvaxia poses a risk of antibody-dependent enhancement (ADE) in seronegative individuals upon subsequent natural infections. This phenomenon heightens the likelihood of severe disease and hospitalization (Fatima and Syed, 2018). As a result, the World Health Organization (WHO) and other regulatory authorities recommend pre-vaccination screening to confirm prior dengue exposure. Usage of Dengvaxia is currently limited to individuals aged 9–45 years residing in dengue-endemic areas with a documented history of previous infection (Marti et al., 2024).

To address existing limitations, Qdenga (TAK-003), developed by Takeda Pharmaceuticals, represents a second-generation live attenuated dengue vaccine. It utilizes a DENV-2 backbone, incorporating the prM and E genes of DENV-1, DENV-3, and DENV-4 to achieve tetravalent protection (Hadinegoro et al., 2015). Unlike Dengvaxia, Qdenga has demonstrated protective efficacy in both seropositive and seronegative individuals, making it viable for wider usage without the need for pre-vaccination screening (Hadinegoro et al., 2015; Tricou et al., 2020). Findings from the TIDES study (Tetravalent Immunization against Dengue Efficacy Study) revealed an overall vaccine efficacy of 80.2% against virologically confirmed dengue and 90.4% efficacy in preventing hospitalization over an 18 months follow-up period. Additionally, Qdenga has shown consistent and durable immunogenicity across different age groups and regions, maintaining an acceptable safety profile with no increased risk identified in dengue-naïve populations (Tricou et al., 2020; Flacco et al., 2024). Nevertheless, considerable challenges persist for both vaccines, including variability in efficacy between serotypes, logistical hurdles in deployment within low-resource settings, and the complexity of integrating them into national immunization programs. Dengvaxia suffered a significant loss of public trust due to adverse outcomes in seronegative children, resulting in program suspensions in several countries (Fatima and Syed, 2018). Conversely, Qdenga has recently gained approval from the European Medicines Agency (EMA) and other regulatory bodies, offering hope for expanded global distribution in dengue-endemic regions without the need for prior serological testing. Ongoing real-world monitoring and cost-effectiveness evaluations will be critical to optimizing the worldwide implementation of dengue vaccination strategies (Flacco et al., 2024; Martinez et al., 2015).

Symptoms typical of the infection, including a petechial rash, myalgia, arthralgia, retro-orbital pain, and a severe headache, are frequently present during this febrile phase. The necessity of attentive hydration and supportive care is highlighted by the fact that dehydration in children during this time might increase the risk of neurological disorders, including seizures (WHO Guidelines Approved by the Guidelines Review Committee, 2009). Because DENV infections are systemic, they can impact several organ systems, making early detection and treatment essential to avoiding serious consequences including DHF and DSS. The introduction of vaccines is a complement to current preventive efforts, such as community awareness programs, vector control methods like the use of insecticides, and the elimination of mosquito breeding sites. The integration of these techniques into national immunization programs and achieving optimal vaccine coverage, however, continue to be critical challenges. Reducing the worldwide burden of DF requires ongoing research into the pathophysiology of DENV and improving the effectiveness of vaccines (Henchal and Putnak, 1990).

## 2 Neurological complications

Neurological complications arising from DENV infection can be grouped into three distinct pathophysiological categories based on the underlying mechanism of injury. The first category involves direct neuroinvasion, characterized by viral entry and replication within the central nervous system (Solomon et al., 2000). The second includes systemic complications, which stem from metabolic disturbances, vascular damage, or hemodynamic instability, occurring without direct invasion of the CNS (Murthy, 2010). The third category encompasses immune-mediated complications, driven by post-infectious autoimmune responses (Carod-Artal et al., 2013). The first is direct neuroinvasion, which is caused by the neurotropism of the virus and causes diseases including encephalitis, myelitis, and meningitis. Serotypes of DENV-2 and DENV-3 are commonly linked to serious neurological outcomes (Garg et al., 2015). Systemic consequences, including strokes, hypokalemic paralysis, and encephalopathy, occur in the second group. These are frequently brought on by severe disease progression and subsequent systemic inflammation. Finally, there is growing recognition of immune-mediated post-infectious consequences, such as acute disseminated encephalomyelitis (ADEM), neuromyelitis optica, and Guillain-Barré syndrome (GBS) (Trivedi and Chakravarty, 2022).

Recent research highlights that environmental variables, host immune responses, and virus serotype all affect these consequences, with endemic areas reporting increased incidence. A almost universal symptom of DF, headaches can be quite severe and are frequently accompanied by phonophobia, photophobia, nausea, and vomiting. In certain instances, headaches are linked to symptoms of lymphocytic meningitis, including positive Kernig’s and Brudzinski’s signs and nuchal stiffness. Interestingly, there is not a difference in the intensity of neurological symptoms between primary and secondary infections or between DHF and DF (Sil et al., 2017).

Early identification and categorization of these issues have been made easier by developments in diagnostic methods, such as neuroimaging and cerebrospinal fluid (CSF) analysis. Inflammatory cytokines and viral RNA levels are two examples of biomarkers that are being investigated for their potential to predict the severity and course of disease. In terms of treatment, supportive care is still critical, especially for immune-mediated problems that usually go away in a few weeks to months (Kothur et al., 2016). However, due to the immediate need to reduce the worldwide neurological burden of dengue, experimental therapies that target inflammation and virus replication are being investigated. Urbanization and climate change are contributing factors to the increasing prevalence of DENV infection, which emphasizes the need of public health initiatives. These include improving immediate diagnosis, vector control, and surveillance, especially in endemic regions. There is potential for lowering morbidity and improving patient outcomes with more investigation into the pathophysiology of DENV’s neurological effects and the creation of focused therapies. With dengue’s neurological symptoms accounting for a substantial portion of disease morbidity, a complete approach is essential as the disease continues to emerge as a major worldwide health concern (Enitan et al., 2024) (Table 1).

**TABLE 1:** Neurological manifestations of dengue virus (DENV) infection.

Category	Neurological manifestation	Mechanism	Clinical description	Diagnostic tools	References
Direct neuroinvasion	Meningitis	Direct viral invasion of CNS Caution: Up to 20% of lumbar punctures may result in peripheral blood contamination, affecting CSF interpretation	Neck stiffness, photophobia, positive Kernig/Brudzinski signs	CSF analysis (lumbar puncture), CSF PCR and serology, Brain MRI, CT scan (pre-LP), EEG, (rarely) brain biopsy	Janssen et al., 1998; Madi et al., 2014
	Encephalitis	Viral replication, cytokine-mediated damage (CSF results must be interpreted carefully due to risk of contamination during lumbar puncture)	Seizures, altered consciousness, focal deficits	Brain MRI, CSF analysis, EEG, viral PCR	Henchal and Putnak, 1990; Madi et al., 2014; Kayesh and Tsukiyama-Kohara, 2022
	Myelitis/acute transverse myelitis	Direct CNS invasion or para-infectious	Sensory and motor deficits, MRI shows spinal cord edema	Spinal MRI, CSF analysis, viral serologies	Carod-Artal et al., 2013; Vieira et al., 2025
	Myositis	Viral muscle invasion, cytokine effect	Muscle pain, elevated CPK; may lead to weakness	Serum CPK, EMG, muscle MRI, muscle biopsy (rarely)	Dalugama et al., 2017; Ghosh et al., 2023; Kaur and Singh, 2021
	Cerebellar Syndrome	Viral neurotropism or inflammatory response	Ataxia, nystagmus; usually self-limited	Neurological exam, brain MRI, optional CSF	Noisakran et al., 2009; Pichl et al., 2022; Trung and Wills, 2010
	Myalgia	Cytokine-mediated inflammation	Diffuse muscle pain without weakness; normal EMG	Clinical assessment, normal CPK, normal EMG	Trivedi and Chakravarty, 2022; Dalugama et al., 2017; Patel et al., 2024
Systemic complications	Encephalopathy	Shock, liver/renal dysfunction, metabolic derangements	Confusion, reduced consciousness; no inflammation	Blood tests (LFT, RFT), EEG, CT/MRI to rule out structural causes	Murthy, 2010; Roberts et al., 2024
	Hypokalemic paralysis	Potassium shift, renal loss, fluid therapy	Flaccid quadriparesis, areflexia; responds to K^+^	Serum electrolytes, ECG, EMG (if needed)	Garg et al., 2015; Akhtar et al., 2023; Malhotra and Garg, 2014
	Stroke	Due to vasculopathy, thrombocytopenia, or coagulopathy not direct CNS invasion	Hemiparesis, cranial nerve deficits; infarct on MRI	Brain MRI/CT, vascular imaging (MRA/CTA), coagulation panel	Trung and Wills, 2010; Verma et al., 2013
	Rhabdomyolysis	Cytokine-induced muscle breakdown	Myalgia, dark urine, elevated CPK; may lead to AKI	Serum CPK, renal function tests, urinalysis (myoglobinuria)	Pichl et al., 2022; Trung and Wills, 2010; Patel et al., 2024
Immune-mediated post-infectious	Guillain-Barré syndrome (GBS)	Autoimmune targeting of peripheral nerves	Progressive weakness, areflexia; may require IVIg	Nerve conduction studies (NCS)/EMG, CSF (albuminocytologic dissociation)	Trivedi and Chakravarty, 2022; Vieira et al., 2025; Carod-Artal, 2014
	ADEM	Demyelinating autoimmune response	Multifocal deficits, seizures, altered sensorium	Brain MRI, CSF analysis, EEG	Garg et al., 2015; Araújo et al., 2012; Chakraborty et al., 2024
	Optic Neuritis	Immune-mediated inflammation of optic nerve	Vision loss, pupillary defect, scotomas	Visual evoked potentials (VEP), brain/orbit MRI, fundus exam	Araújo et al., 2012; Jugpal et al., 2017; Lim et al., 2023
	Neuromyelitis optica	Autoantibody-mediated demyelination	Involves optic nerves and spinal cord	AQP4 antibody testing, spinal/brain MRI, CSF analysis	Carod-Artal et al., 2013; Garg et al., 2015
	Parkinsonism	Post-viral basal ganglia inflammation	Rigidity, tremor, bradykinesia; may be transient	Clinical exam, DaT scan (if available), brain MRI	Garg et al., 2023; Hopkins et al., 2022
	OMAS (opsoclonus-myoclonus-ataxia syndrome)	Autoimmune cerebellar dysfunction	Chaotic eye movements, myoclonus, ataxia	Clinical diagnosis, brain MRI, CSF (sometimes)	Garg et al., 2023
	Cranial Neuropathies (e.g., oculomotor, thoracic)	Post-infectious immune targeting	Diplopia, ptosis, facial palsy, shoulder droop	Brain MRI, EMG/NCS if needed, clinical exam	Trivedi and Chakravarty, 2022; Pichl et al., 2022; Trung and Wills, 2010

### 2.1 Direct neuroinvasion

A form of “direct neuroinvasion” describes the DENV capacity to enter the CNS directly, leading to neurological complications, including encephalitis, meningitis, myelitis, and myositis. These conditions arise from inflammation triggered directly by viral replication within neural tissues, setting them apart from systemic or autoimmune inflammatory responses. The main characteristic of these conditions is inflammation of the brain, spinal cord, and related tissues, which can have severe consequences and, in certain situations, cause irreversible neurological damage. Although the exact processes by which the DENV enters the CNS remain unclear, an increasing amount of data indicates that the virus’s direct invasion of neural tissue plays a major role in the emergence of these conditions. Meningitis, encephalitis, and myelitis are the most often seen neurological symptoms in individuals with direct neuroinvasion of dengue. While encephalitis includes direct inflammation of the brain tissue itself, meningitis is defined by inflammation of the protective membranes surrounding the brain and spinal cord (Farina et al., 2023). Acute transverse myelitis manifests as spinal cord inflammation, while myositis is defined as inflammation of muscle tissue. Cerebellar syndrome and myalgia can also appear; these conditions often show up as issues with coordination and muscular pain, respectively. These clinical manifestations demonstrate the wide range of neurological harm that can arise from a DENV infection of the CNS (Kondziella and Waldemar, 2023).

The DENV capacity to cause CNS infections depends on its capacity to pass across the blood-brain barrier (BBB), a protective barrier that typically keeps pathogens from entering the brain. This is especially significant during the infection’s acute phase, when the majority of neurological problems appear. According to research, the virus can either directly infiltrate neurons or cause immune-mediated inflammation, which has an indirect effect on the CNS. Studies using animal models have shed important light on how the virus causes the production of cytokines that promote inflammation, which may compromise the BBB. When this barrier breaks down, the virus can enter the brain and spinal cord, where it can multiply and intensify the inflammatory reaction (Mustafá et al., 2019). Research involving spinal cord injury models has demonstrated that enhancing autophagy while suppressing necroptosis can reduce neural damage. This indicates that strategic modulation of these pathways might also offer potential for addressing DENV-induced neuroinflammation and central nervous system injury (Chen et al., 2025). The hypothesis of direct neuroinvasion has been strongly supported by recent histological investigations. Histological evidence of encephalitis, including the presence of inflammatory cells in brain tissue, was found during the autopsy of a child who had been diagnosed with dengue encephalitis. Further evidence of the virus’s capacity to impact the CNS comes from the isolation of DENV types 2 and 3 from CSF samples taken from individuals exhibiting neurological symptoms. The theory that the virus can directly infect neural tissue is further supported by the discovery of dengue antigens in the brain tissue of affected individuals, in addition to the virus’s isolation. The pathophysiology of dengue-related encephalitis has been studied by researchers as a result of these outcomes, with an emphasis on the mechanisms that allow the virus to enter the CNS and the following effects it has on brain function (Ludlow et al., 2016).

Numerous cytokines, including interleukins and interferons, have been demonstrated to be activated as part of an immune response triggered by DENV infection in animal models. These cytokines play a key role in modulating the inflammatory response, and the BBB may become more permeable as a result of their high levels in the CSF. This increased permeability could make it easier for the virus to enter the central nervous system, starting the series of events that eventually result in myelitis, encephalitis, and other neurological disorders. Despite being intended to manage the infection, this inflammatory reaction may severe damage the nerve system and leading long-term neurological consequences in affected patients (Guabiraba and Ryffel, 2014).

Most cases of myositis associated with dengue fever tend to be self-limiting, resolving effectively with supportive care alone (Dalugama et al., 2017). This condition, marked by inflammation of muscle tissue, typically manifests through symptoms such as muscle tenderness, weakness, and elevated levels of creatine phosphokinase (CPK) (Trivedi and Chakravarty, 2022). Early diagnosis is crucial to avoid severe complications, making CPK assessment and urinalysis key diagnostic measures for dengue patients exhibiting muscle-related symptoms. For cases where dengue myositis persists, corticosteroid therapy has demonstrated notable clinical benefits (Kaur and Singh, 2021).

**FIGURE 1:**
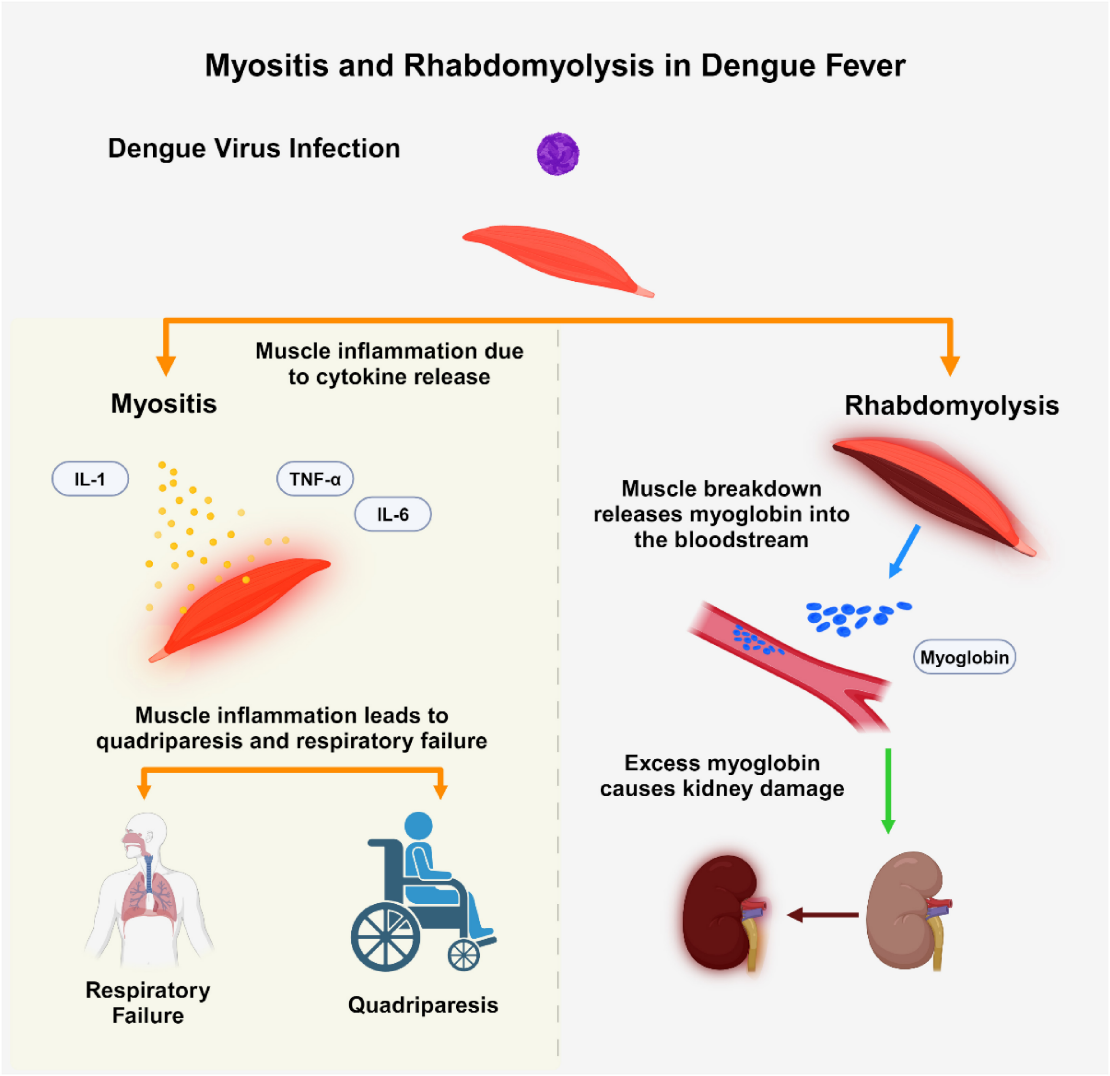
This figure illustrates two muscle-related complications in dengue fever: myositis and rhabdomyolysis. Dengue virus (DENV) infection can induce myositis through cytokine release (IL-1, IL-6, TNF-α), resulting in muscle inflammation, quadriparesis, and respiratory failure. Conversely, rhabdomyolysis is characterized by muscle breakdown, leading to myoglobin release into the bloodstream, potentially causing acute kidney injury (AKI) due to myoglobin accumulation. The Figure was designed using BioRender.com.

Conversely, myelitis especially acute transverse myelitis is an uncommon but serious neurological complication tied to dengue infection (Carod-Artal et al., 2013). This form of spinal cord inflammation may develop during the active phase of the disease due to direct viral invasion (para-infectious) or in the post-infectious phase as an immune-mediated response (Vieira et al., 2025). Patients with myelitis often present with limb weakness, sensory impairment, and difficulties in bowel or bladder function. Diagnosis typically involves spinal MRI, which may reveal edema or signal changes, alongside CSF analysis that could indicate the presence of viral RNA or intrathecal IgG production (Verma et al., 2013; Vieira et al., 2025; Figure 1).

The rare and serious neurological side effect of dengue infection is cerebellar involvement. Between 2 days and 2 weeks after the start of the DENV, this disease usually appears (Trung and Wills, 2010). Although they are still not fully understood, the pathophysiological processes generating cerebellar involvement appear to vary among patients. Direct viral invasion of the CNS and an inflammatory reaction brought on by the infection are two hypothesized processes (Noisakran et al., 2009). Viral neurotropism, in which the virus directly damages cerebellar tissues, is considered to be linked to the early development of cerebellar symptoms. In contrast, the inflammatory response, which takes longer to develop, may contribute to delayed symptom presentation. Key clinical features of cerebellar syndrome in DF include ataxia and bilateral nystagmus, which highlight the dysfunction of cerebellar pathways. However, due to the limited characterization of the association between cerebellar syndrome and DENV, it is critical to perform a thorough differential diagnosis to exclude other potential etiologies. Magnetic resonance imaging (MRI) of the brain is often used in diagnostic evaluation, yet findings are typically unremarkable, with normal scans reported in most documented cases. Notably, dengue fever-related cerebellar syndrome often has a benign course. Patients usually fully recover without any lasting neurological impairments, and it is often transient and self-limiting. This good prognosis highlights the significance early detection and supportive care are to symptom management and the best possible recovery results. To further understand the processes, risk factors, and therapeutic care approaches for this uncommon issue, more research is necessary (Pichl et al., 2022).

In the early stages of dengue fever, myalgia is a common and distinct symptom that indicates the disease’s systemic involvement (Trivedi and Chakravarty, 2022). It is believed that both direct viral invasion of muscle tissues and inflammatory alterations caused on by the immune system’s reaction to the infection are the underlying processes causing muscular soreness (Dalugama et al., 2017). The typical symptoms of dengue-associated myalgia are probably caused by this combination. These symptoms cause a great deal of pain and mostly impact the back and proximal muscle groups. Dengue-associated myalgia can be distinguished from other disorders affecting muscle dysfunction because to this differentiation. Despite the severity of the pain, myalgia associated with DF is often transient and self-limiting, with symptoms going away once the disease’s acute phase subsides. This benign course emphasizes the value of supportive care, which aims to reduce pain and guarantee that patients heal without suffering from long-term muscle damage. Deeper understanding of the mechanism and the best ways to treat this prevalent DF presentation may be possible with more study (Patel et al., 2024).

### 2.2 Systemic complications

This category includes conditions caused by widespread body responses to the viral infection, which indirectly impact CNS function. Examples include encephalopathy (brain dysfunction without inflammation), hypokalemic paralysis (paralysis due to low potassium levels), and stroke. These complications result from metabolic disturbances, dehydration, or other systemic effects of the infection (Murthy, 2010). Encephalopathy is the most frequently observed neurological manifestation of DENV infection. It arises due to systemic complications that occur with severe infection, such as shock, metabolic imbalances, cerebral edema, and organ dysfunction involving the liver or kidneys (Roberts et al., 2024). DENV encephalitis frequently presents with seizures, along with altered mental status, headaches, behavioral changes, and various focal neurological deficits (Araújo et al., 2012; Domingues et al., 2008). Neuroimaging of patients with encephalitis typically reveals T2-weighted and fluid-attenuated inversion recovery (FLAIR) hyper intensities. These hyper intensities are primarily found in deep brain structures, with the thalamus, basal ganglia, and cerebellum being commonly affected (Jugpal et al., 2017).

Intracranial hemorrhagic complications in dengue infection can be attributed to a bleeding diathesis resulting from vasculopathy, thrombocytopenia, and platelet dysfunction (Trung and Wills, 2010). Thrombocytopenia in dengue arises from both reduced production and increased destruction of platelets, with its severity often correlating to the clinical intensity of DHF and activation of the complement system (Noisakran et al., 2009). This impairment in platelet function elevates the risk of vascular fragility and subsequent hemorrhage. Additionally, dengue-related coagulopathy and vasculopathy can contribute to vascular thrombosis and ischemic stroke. Elevated levels of plasminogen activator inhibitor type I (PAI-I), a procoagulant, are also observed in dengue patients, further impacting coagulation dynamics (Trung and Wills, 2010). In a case study, a 68 years-old man with dengue presented with left-sided facial paresis and hemiparesis. Blood tests showed leukocytosis and thrombocytopenia, and dengue NS1 antigen was positive. CSF analysis was normal except for 15 lymphocytes. MRI indicated an acute infarct in the right parietal region. After conservative treatment with low-dose aspirin and physiotherapy, he showed partial improvement in limb strength at a 2 months follow-up (Verma et al., 2013).

Hypokalemic paralysis, a disorder marked by a sudden onset of muscular weakness caused on by critically low blood potassium levels, has been linked to DENV infection. It is difficult to comprehend the pathophysiology of hypokalemia in relation to DF since this electrolyte imbalance is caused by several overlapping pathways. A number of theories provide insight into the possible causes and interactions, even if exact pathways are yet unknown (Garg et al., 2013). The disease known as metabolic alkalosis, in which the blood becomes too alkaline, can result from these fluids. Despite being a well-established physiological reaction, this intracellular shift is especially relevant in dengue because of the intensive fluid resuscitation techniques frequently used to treat severe dehydration or dengue shock syndrome. The condition is made more difficult by the systemic nature of dengue. As part of the more extensive inflammatory and metabolic abnormalities observed in severe cases, the infection may result in a redistribution of potassium between cells and extracellular fluid. These renal abnormalities might be the direct result of the virus’s impact on kidney function or a secondary outcome of changes in vascular permeability and systemic inflammation. Hypokalemia may worsen as a result of increased catecholamine levels stimulating cellular potassium absorption through beta-adrenergic receptors. Although this method inadvertently leads to potassium depletion, it represents the body’s attempt to counteract the systemic stress brought on by the disease. At the molecular level, ion channel-related genetic predispositions have also been linked to hypokalemic paralysis in dengue (Garg et al., 2015). This interaction raises the possibility that certain people are genetically more likely to experience hypokalemia in response to particular stresses, such as dengue illness. The intricate link between systemic inflammation, metabolic disorders, renal dysfunction, and genetic predispositions is highlighted by the multifaceted character of hypokalemia in dengue. Similar to observations in ischemic tissue models, where SNAP29-driven disruptions in autophagic processes and parthanatos exacerbate mitochondrial damage, these mechanisms may likewise play a role in neuronal or muscular injury during severe dengue, potentially driven by oxidative stress and imbalances in cellular homeostasis. It is essential to comprehend these pathways in order to enhance therapeutic management (Yang et al., 2025). Timely intervention, such as cautious fluid control, potassium supplementation, and careful monitoring of renal function and electrolyte balance, is made possible by early detection of hypokalemia and its underlying causes. In order to manage hypokalemic paralysis linked to DENV infection, further study is required to examine the genetic variables and molecular pathways involved. This might lead to the development of individualized treatment plans (Harraz and Delpire, 2024).

Dengue patients complicated with hypokalemic paralysis may present as pure motor weakness of all four limbs (Garg et al., 2015). The quadriparesis can be accompanied by areflexia, hyporeflexia, and hypotonia. Hypokalemic paralysis generally occurs between 2 and 5 days after the onset of fever. A period of 4–24 h of weakness is witnessed. The development of weakness was usually manifested in the phase of defervescence of the febrile stage of dengue. Dengue-associated hypokalemic paralysis must be suspected in patients with motor weakness in dengue-endemic areas. Acute onset of flaccid quadriplegia without any cranial nerve palsy and without any impaired compromise should take our attention to hypokalemic paralysis (Malhotra and Garg, 2014). The serum potassium levels of all suspected Guillain-Barré syndrome patients must be checked. When the serum potassium reaches to 3 mmol/liter or below, the diagnosis of hypokalemic paralysis is indicated. Dengue-associated hypokalemic paralysis is treated completely with adequate potassium supplementation. The patients evolve a rapid recovery without any residual weakness (Akhtar et al., 2023).

### 2.3 Immunological post-infectious complications

This group comprises complications that emerge after the primary infection has resolved, triggered by the body’s immune response to the virus (Murthy, 2010). Conditions in this category include encephalomyelitis (widespread inflammation of the brain and spinal cord), GBS (an autoimmune disorder causing muscle weakness), optic neuritis (inflammation of the optic nerve), Mononeuropathy, ADEM, Brachial neuritis, Cerebellitis, Opsoclonus–myoclonus–ataxia syndrome, Parkinsonism, neuromyelitis optica (inflammation of the optic nerve and spinal cord), and cranial neuropathies such as oculomotor and long thoracic nerve palsies. These immune-mediated complications typically develop weeks to months after infection and often resolve gradually over time (Trivedi and Chakravarty, 2022).

**FIGURE 2:**
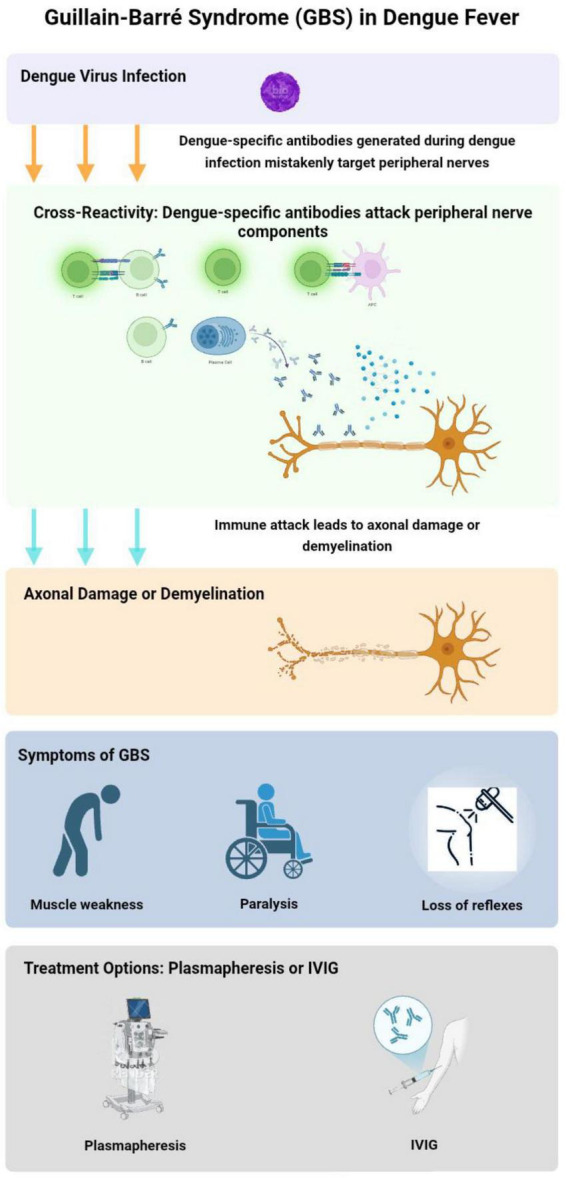
Pathogenesis of Guillain-Barré syndrome (GBS) in dengue fever. This flowchart illustrates the development of GBS during dengue fever. The process begins with dengue virus (DENV) infection, leading to immune cross-reactivity where viral antibodies attack peripheral nerve components. This results in axonal damage or demyelination, manifesting as muscle weakness, paralysis, and loss of reflexes Treatment options include plasmapheresis and intravenous immunoglobulin (IVIg) therapy to reduce immune-mediated nerve damage. The Figure was designed using BioRender.com.

Guillain-Barré syndrome can occur either early or late in the progression of the illness. When immunoglobulins induced by DF interact with components of peripheral nerves that share cross-reactive epitopes, they may trigger an immune response. This response can damage axons or myelin, leading to axonal damage and demyelination, which results in polyneuropathy (Trivedi and Chakravarty, 2022). GBS cases emerge following the acute phase of DF, suggesting an autoimmune mechanism as the underlying cause (Vieira et al., 2025). Both DF and GBS are thought to involve similar pro-inflammatory mediators, potentially triggering the onset of GBS after dengue infection (Sil et al., 2017; Pandey et al., 2018). Key factors in this process include interleukins, tumor necrosis factor-alpha, and complement proteins, all of which may contribute to GBS pathogenesis post-dengue. Additionally, due to a cross-immune response, the body’s immune cells can mistakenly target the myelin and axons of spinal cord roots, leading to nerve damage (Carod-Artal et al., 2013; Figure 2).

A case reported by Lim et al. (2023) in June 2023 involved a 49 years-old man with a known history of type 2 diabetes and dyslipidemia, who presented with symmetrical weakness of the lower limbs just 2 days after the onset of DF (febrile phase). This presentation is uncommon, as other case studies indicate that GBS typically occurs 1 or 2 weeks after the onset of DF (Lim et al., 2023).

**FIGURE 3:**
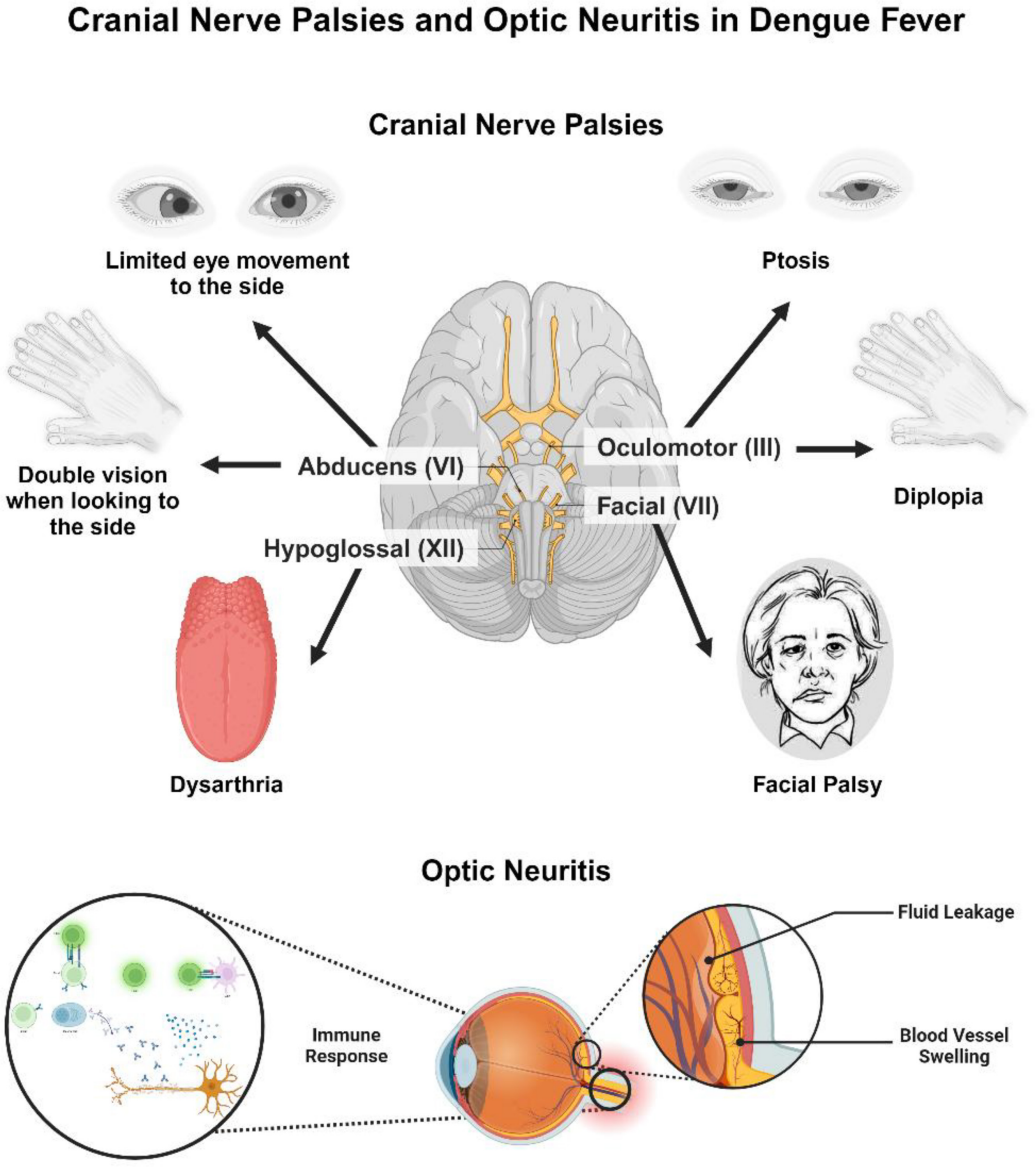
Cranial nerve palsies and optic neuritis in dengue fever. This figure illustrates cranial nerve involvement and optic neuritis as complications of dengue fever. Affected cranial nerves include the oculomotor nerve (III), abducens nerve (VI), facial nerve (VII), and hypoglossal nerve (XII), leading to symptoms such as ptosis, diplopia, limited lateral eye movement, facial palsy, and dysarthria. The lower section depicts optic neuritis, with immune-mediated inflammation of the optic nerve, leading to vascular leakage and vasculitis. The immune response contributing to this inflammation is also depicted, indicating the underlying pathophysiological mechanisms. The Figure was designed using BioRender.com.

Cranial nerve involvement includes oculomotor nerve palsy, optic neuritis, long thoracic neuropathy and isolated palsies of sixth and seventh and phrenic nerves (Trivedi and Chakravarty, 2022; Trung and Wills, 2010). Optic neuritis may occur post-dengue fever, with symptoms like decreased vision, weakened color perception, pupillary defects, and scotomas. Although optic neuritis typically affects both eyes, some patients may experience unilateral symptoms (Noisakran et al., 2009). The exact cause of post dengue optic neuritis after is unclear, but it may involve complex vascular leakage (Araújo et al., 2012). The condition may result from direct viral infection or immune-mediated responses following the illness (Garg et al., 2015). The delayed onset of visual symptoms likely favors an immune-mediated process over direct viral infection. However, the precise pathogenesis is still undetermined. Affected patients generally present with blurred vision, weakened color perception, pupillary defects, and scotomas. Both eyes are usually involved, but some patients may show unilateral symptoms. Early recognition is essential to prevent permanent ocular complications (Jugpal et al., 2017; Lim et al., 2023). Differentiating between localized inflammation caused by direct viral replication in the central nervous system, such as in encephalitis or meningitis, and systemic or immune-mediated inflammation, seen in conditions like encephalopathy or Guillain-Barré Syndrome, is crucial to prevent diagnostic confusion. This distinction highlights their unique underlying mechanisms and plays a vital role in guiding clinical decisions (Ellul and Solomon, 2018). Ocular manifestations of dengue are managed symptomatically, often involving the use of anti-inflammatory and immunosuppressive agents. However, it remains uncertain whether observed improvements are due to therapeutic interventions or the natural course of recovery. The primary focus of treatment is supportive care, with corticosteroids showing potential benefits in the early stages of the disease (Wang et al., 2024; Figure 3).

### 2.4 Acute disseminated encephalomyelitis (ADEM)

Acute disseminated encephalomyelitis is an uncommon inflammatory demyelinating disease of the CNS that usually progresses in a monophasic course and is characterized by multifocal white matter involvement. ADEM frequently appears during the recovery stage of a dengue disease. Seizures, focal neurological abnormalities, and altered awareness are possible clinical manifestations; they often happen after the febrile phase has passed (Yip et al., 2012). The underlying pathophysiology is a temporary autoimmune response that targets unknown self-antigens or myelin. Diagnostic techniques like CSF analysis and brain MRI can yield important information. Methylprednisolone administered by pulse intravenously has shown therapeutic effectiveness during the active period of ADEM. Two weeks after an uncomplicated dengue episode, a 38 years-old male developed ADEM, according to a noteworthy case documented by Chakraborty et al. (2024). This uncommon condition was characterized by lower limb weakness and numbness, bowel and bladder incontinence, and a band-like feeling in the T4 dermatome. Importantly, no indications of cerebral involvement were seen. The brain and spinal cord have demyelinating lesions, according to imaging scans. Intravenous corticosteroids were used to treat the patient with success, highlighting the need of quick action in treating this uncommon dengue-related disease (Chakraborty et al., 2024).

**FIGURE 4:**
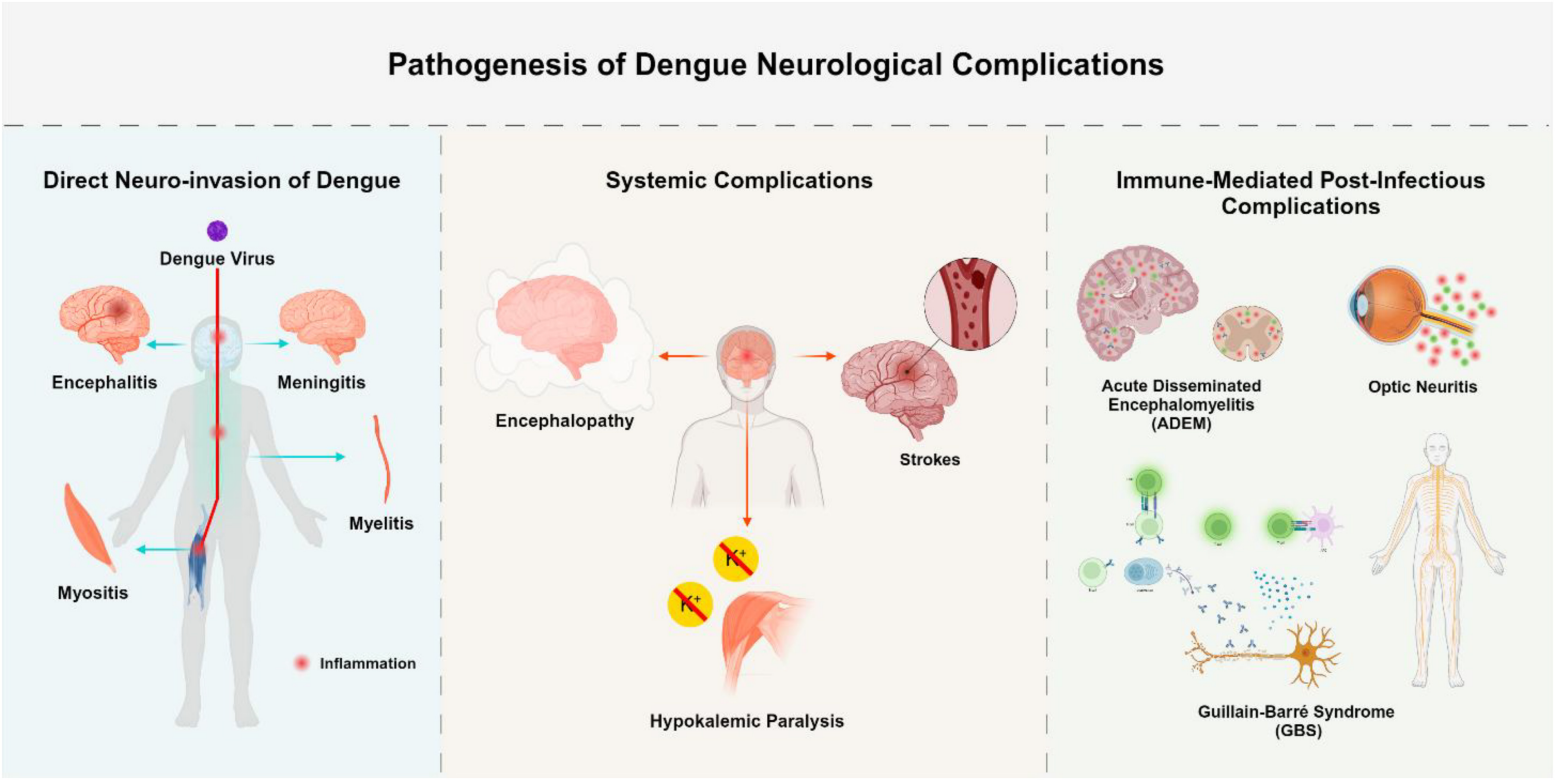
Pathogenesis of dengue neurological complications. This figure summarizes the neurological complications of dengue virus (DENV) based on three pathophysiological pathways: Direct Neuroinvasion [via viral entry and localized central nervous system (CNS) inflammation due to viral replication]; Systemic Complications (via indirect mechanisms such as vasculopathy, coagulopathy, or metabolic disturbance); Immune-Mediated Complications (via post-infectious immune responses leading to demyelination or neuronal injury). The Figure was designed using BioRender.com.

Bradykinesia, tremor, postural instability, and stiffness are the four cardinal signs of Parkinsonism that are present after viral infections. Inflammation-induced disruption of dopaminergic transmission is thought to be the etiology of viral parkinsonism. The development of parkinsonism during or after the course of dengue disease and clinical evidence of dengue infection verified by laboratory testing are diagnostic criteria for dengue-associated parkinsonism. The majority of individuals with this condition are men, according to a research, and they exhibit lymphocytosis, speech problems, and an emotionless facial expression (Hopkins et al., 2022). Opsoclonus-myoclonus ataxia syndrome (OMAS) is a neurological disorder characterized by involuntary eye movements and muscle spasms. Its pathophysiology involves autoimmune dysfunction targeting Purkinje cells in the cerebellar dorsal vermis. This leads to motor control disruption by disinhibiting the oculomotor fastigial region and impairing omnipause cells in the pontine raphe nucleus. Neurological complications of dengue-related OMAS can be categorized into direct neurotropic effects, systemic neurological effects due to metabolic disturbances, and immune-mediated effects such as ADEM, GBS, and OMAS itself. Treatment often includes high-dose intravenous methylprednisolone followed by tapered oral prednisolone (Garg et al., 2023). Numerous viruses, such as the measles, dengue, Epstein-Barr virus, and varicella-zoster virus, can produce cerebellitis, a consequence of viral encephalitis. Ataxia may be the outcome of immune-mediated cerebellar dysfunction or direct viral invasion. There have only been five cases of dengue-associated cerebellitis documented in 2018. With the exception of one instance, which had T2 hyperintensity in the cerebellum, the majority of patients had normal brain MRIs. Another report noted a “bright MCP sign” with T2-weighted hyperintensities in the cerebellar peduncle, successfully treated with intravenous methylprednisolone (Costa and Sato, 2020). Diagnostic and imaging tools for neurological manifestations shown in Table 2. The neurological manifestations of DENV infection are shown in Figure 4.

**TABLE 2:** Neurological manifestations of dengue: pathophysiology and clinical features.

Condition	Pathophysiology	Clinical notes	Pathophysiology references	Clinical references
Meningitis	Inflammation of the meninges due to direct CNS invasion	Presents with neck stiffness and positive Kernig/Brudzinski signs	(Madi et al., 2014; Mustafá et al., 2019)	Janssen et al., 1998; Soares et al., 2010; Goswami et al., 2012
Headache	Cytokine-mediated neuroinflammation	Frontal or retro-orbital pain, bilateral throbbing; with photophobia, nausea	(Trivedi and Chakravarty, 2022; Patel et al., 2024)	da Fonseca and Fonseca, 2002; Domingues et al., 2006
Encephalitis	Viral replication and cytokine-induced neuronal damage	Seizures, altered consciousness, focal deficits; more common in DENV-2/3	(Kayesh and Tsukiyama-Kohara, 2022; Ludlow et al., 2016; Guabiraba and Ryffel, 2014)	Henchal and Putnak, 1990; Madi et al., 2014; Jugpal et al., 2017
Acute transverse myelitis	Immune-mediated demyelination or direct viral insult	Weakness, sensory loss, spinal edema on MRI	(Verma et al., 2013; Vieira et al., 2025)	Verma et al., 2013; Carod-Artal et al., 2013; Vieira et al., 2025
Hypokalemic paralysis	Potassium shift, renal tubular dysfunction, catecholamine surge	Occurs during defervescence; flaccid quadriparesis, rapid recovery	(Garg et al., 2015; Harraz and Delpire, 2024)	Akhtar et al., 2023; Malhotra and Garg, 2014
Guillain-Barré syndrome (GBS)	Autoimmune demyelination of peripheral nerves	Progressive weakness, areflexia, sensory loss	(Carod-Artal et al., 2013; Trivedi and Chakravarty, 2022; Carod-Artal, 2014)	Vieira et al., 2025; Lim et al., 2023
Rhabdomyolysis	Cytokine-mediated muscle breakdown; Ca^2+^ dysregulation	Myalgia, dark urine, elevated CPK; risk of acute kidney injury	(Patel et al., 2024; Guabiraba and Ryffel, 2014)	Noisakran et al., 2009; Pichl et al., 2022

## 3 Management

DF continues to lack a specific antiviral treatment, necessitating primarily supportive care strategies (Trivedi and Chakravarty, 2022). These approaches aim to stabilize the patient by focusing on meticulous fluid management, blood pressure monitoring, and fever reduction. Vigilant hematological surveillance is essential to identify complications, such as thrombocytopenia or hemoconcentration, which are characteristic of severe cases. For patients displaying neurological manifestations, a thorough clinical evaluation is crucial to discern potential contributing factors, including electrolyte disturbances, intracranial hemorrhages, and organ failures such as hepatic or renal insufficiency (Patel et al., 2024). Management of neurological symptoms associated with DF involves airway protection in patients with altered mental status or seizures, ensuring adequate hydration and nutrition, and careful observation of consciousness levels. Because non-hepatotoxic anti-epileptic drugs reduce the risk of liver damage in a weakened hepatic environment, their usage is recommended for the management of seizures (Kakde and Khatib, 2024). The effectiveness of corticosteroids and antiviral drugs for severe neurological complications like dengue encephalopathy or encephalitis is still unknown, especially in critical care settings when these treatments have not demonstrated clear advantages (Prabhat et al., 2020). Both direct invasion of the CNS and ADE, which increases the immune response, are ways that the DENV causes neurological damage. Supportive treatments, such as careful monitoring of awareness, airway protection, and seizure management, are therefore designed to meet the particular neurological signs. Appropriate measures must be taken when there is high intracranial pressure. While there is currently little evidence, IVIg with pulsed steroids may provide therapeutic advantages for immune-mediated disorders such as GBS or ADEM. Treatments for GBS include plasmapheresis and high-dose IVIg, which are usually given at a dosage of 2 mg/kg to enhance recovery (Carod-Artal et al., 2013; Lim et al., 2023). Neurorehabilitation is crucial for the post-acute management of patients experiencing neurological complications related to dengue, especially when residual motor, cognitive, or sensory deficits are present. Employing strategies such as physical therapy, occupational therapy, and cognitive rehabilitation can greatly improve functional recovery and overall quality of life. While the acute treatment phase mainly focuses on supportive care, seizure management, and immunotherapy, incorporating structured rehabilitation into long-term care plans is essential. This approach is particularly important for addressing conditions like Guillain-Barré syndrome, post-encephalitic cognitive impairment, and ataxia resulting from cerebellitis (Khan and Amatya, 2012; Castell et al., 2025). Management and treatment strategies of neurological manifestation shown in Table 3, and complications of dengue-associated viral encephalitis and their management shown in Table 4.

**TABLE 3:** Management and treatment strategies of neurological manifestation.

Neurological manifestation	Incidence	Prognosis	Treatment	Route	Class of Recommendation/ Level of Evidence	Clinical considerations	Pathophysiology references	Clinical references
Encephalitis	Rare	Variable; can lead to long-term deficits	Supportive care, corticosteroids	IV/Oral	BIII	Requires neuroimaging, seizure control, airway protection	(Kayesh and Tsukiyama-Kohara, 2022; Guabiraba and Ryffel, 2014; Domingues et al., 2008)	Roberts et al., 2024; Araújo et al., 2012; Jugpal et al., 2017
Stroke	Very rare	Depends on extent of vascular damage	Supportive care, antiplatelets	Oral/IV	CIII	Often due to thrombocytopenia and vasculitis; MRI-guided management	(Noisakran et al., 2009; Trung and Wills, 2010; Patel et al., 2024)	Verma et al., 2013
Myositis	Rare	Usually self-limiting	Corticosteroids if persistent	Oral	BII	CPK and urine monitoring; rule out rhabdomyolysis	(Trivedi and Chakravarty, 2022; Dalugama et al., 2017; Guabiraba and Ryffel, 2014)	Ghosh et al., 2023; Kaur and Singh, 2021; Pichl et al., 2022; Gulati et al., 2020
ADEM	Very rare	Favorable with early immunotherapy	High-dose IV steroids	IV	AI	MRI essential; often follows febrile phase	(Carod-Artal et al., 2013; Trivedi and Chakravarty, 2022)	Garg et al., 2015; Chakraborty et al., 2024
Guillain-Barré syndrome	Uncommon	Recovery over weeks to months	IVIg, plasmapheresis	IV	AI	Early intervention critical; rule out hypokalemia	(Carod-Artal et al., 2013; Trivedi and Chakravarty, 2022; Vieira et al., 2025; Carod-Artal, 2014)	Sil et al., 2017; Lim et al., 2023; Pandey et al., 2018

The “Class of Recommendation/Level of Evidence” column reflects a standardized grading system for clinical guidance. The Class of Recommendation (I, II, III) indicates the strength of the recommendation, while the Level of Evidence (A, B, C) reflects the quality of the supporting evidence. Class I: Strong recommendation; benefit > risk. Class II: Moderate recommendation; benefit ≥ risk. Class III: Recommendation not advised; no benefit or harm > benefit. Level A: High-quality evidence from multiple RCTs or meta-analyses. Level B: Moderate-quality evidence from one RCT or nonrandomized studies. Level C: Expert opinion or limited data.

**TABLE 4:** Complications of dengue-associated viral encephalitis and their management.

Complication	Mechanism	Clinical features	Management	Level of Evidence	References
Cerebral edema	Inflammatory cytokine surge, BBB disruption	Altered mental status, increased intracranial pressure	IV mannitol, hypertonic saline, ventilatory support	BIII	Janssen et al., 1998; Roberts et al., 2024; Jugpal et al., 2017
Seizures	Cortical irritation due to viral inflammation	Generalized or focal seizures, post-ictal confusion	Non-hepatotoxic antiepileptics (e.g., levetiracetam)	AII	Trivedi and Chakravarty, 2022; Patel et al., 2024; Kakde and Khatib, 2024
Intracranial hemorrhage	Thrombocytopenia, vasculopathy	Headache, decreased consciousness, focal neurological deficits	Platelet transfusion, conservative neurocritical care	BII	Noisakran et al., 2009; Trung and Wills, 2010; Jugpal et al., 2017
Status epilepticus	Refractory seizure activity from persistent CNS insult	Continuous seizures > 5 min, altered consciousness	IV benzodiazepines, antiepileptic loading, ICU admission	AI	Trivedi and Chakravarty, 2022; Prabhat et al., 2020
Cerebellitis	Direct viral invasion or immune-mediated mechanism	Ataxia, dysarthria, nystagmus	Supportive care, high-dose corticosteroids if immune-mediated	BII	Trivedi and Chakravarty, 2022; Trung and Wills, 2010; Costa and Sato, 2020
Hydrocephalus (rare)	Obstructive CSF flow due to inflammatory exudate	Papilledema, vomiting, altered sensorium	Neurosurgical evaluation, external ventricular drainage if indicated	CIII	Carod-Artal et al., 2013; Trivedi and Chakravarty, 2022
Secondary bacterial superinfection	Nosocomial or iatrogenic complication	Recurrent fever, CSF pleocytosis, elevated CRP	Empirical IV antibiotics (e.g., ceftriaxone ± vancomycin)	AIII	Murthy, 2010; Trivedi and Chakravarty, 2022
Cognitive impairment (post-encephalitic)	Neuronal injury, hippocampal involvement	Memory deficits, attention difficulties, personality changes	Neurorehabilitation, occupational therapy	BIII	Vieira et al., 2025; Chakraborty et al., 2024

## 4 Conclusion

With its complex pathophysiology and neurological consequences that significantly increase disease morbidity, DENV poses a serious public health risk. Through complex processes including direct viral neuroinvasion, cytokine dysregulation, and changes in vascular permeability, the virus can cause neurological symptoms including encephalitis, meningitis, and immune-mediated disorders like GBS. Pathophysiologically, the development of neurological problems depends mainly on DENV’s interactions with the blood-brain barrier, immune response regulation, and systemic inflammation. Although CSF analyses and neuroinflammatory markers have improved our knowledge of these processes, the exact molecular mechanisms are still not well-understood. Effective intervention is limited by the lack of specific antiviral treatments, even with advancements in diagnostics and supportive care techniques. Neurological outcomes further emphasize the critical need for comprehensive methods that include the development of immunomodulatory treatments, new diagnostic tools, and public health measures. Reducing the worldwide neurological burden of DENV and improving patient outcomes will require understanding the complex processes underlying the neurotropism and immunological interactions of the diseases.
